# Genetic diversity and differentiation in narrow versus widespread taxa of *Helianthemum* (Cistaceae) in a hotspot: The role of geographic range, habitat, and reproductive traits

**DOI:** 10.1002/ece3.4481

**Published:** 2019-03-05

**Authors:** Sara Martín‐Hernanz, Sara Martínez‐Sánchez, Rafael G. Albaladejo, Juan Lorite, Juan Arroyo, Abelardo Aparicio

**Affiliations:** ^1^ Departamento de Biología Vegetal y Ecología Universidad de Sevilla Sevilla Spain; ^2^ Departamento de Botánica Universidad de Granada Granada Spain; ^3^ Present address: Departamento de Tecnología de la Alimentación y Nutrición Universidad Católica de Murcia Murcia Spain

**Keywords:** edaphism, endemism, genetic diversity, *Helianthemum*, hotspot, reproductive traits, Sierra Nevada

## Abstract

Unraveling the relationships between ecological, functional traits and genetic diversity of narrow endemic plants provide opportunities for understanding how evolutionary processes operate over local spatial scales and ultimately how diversity is created and maintained. To explore these aspects in Sierra Nevada, the core of the Mediterranean Betic‐Rifean hotspot, we have analyzed nuclear DNA microsatellite diversity and a set of biological and environmental factors (physicochemical soil parameters, floral traits, and community composition) in two strictly endemic taxa from dolomite outcrops of Sierra Nevada (*Helianthemum pannosum* and *H. apenninum* subsp. *estevei*) and two congeneric widespread taxa (*H. cinereum* subsp. *rotundifolium* and *H. apenninum* subsp.* apenninum*) that further belong to two different lineages (subgenera) of *Helianthemum*. We obtained rather unexpected results contrasting with the theory: (a) The narrow endemic taxa showed higher values of genetic diversity as well as higher average values of pollen production per flower and pollen‐to‐ovule ratio than their widespread relatives; and (b) the two taxa of subg. *Helianthemum*, with larger corollas, approach herkogamy and higher pollen production than the two taxa of subg. *Plectolobum*, displayed lower genetic diversity and higher values of inbreeding. Altogether, these results disclose how genetic diversity may be affected simultaneously by a large number of intrinsic and extrinsic factors, especially in Pleistocene glacial refugia in mountains where the spatial context harbors a great ecological heterogeneity. On the other hand, differences in mating system and the significant effect of the substrate profile, both being highly diverse in the genus *Helianthemum*, in the genetic variability illustrate about the importance of these two factors in the diversification and species differentiation of this paradigmatic genus in the Mediterranean and open the field to formulate and test new hypotheses of local adaptation, trait evolution, and habitat diversification.

## 1 INTRODUCTION

It is well known that biological diversity is concentrated in particular regions around the world, so‐called biodiversity hotspots. Despite scanty information being available about the building up of biodiversity in such regions, attention is recently being paid to the evolutionary history of species assemblages and lineages by considering phylogenetic relationships for whole floristic datasets (e.g., Molina‐Venegas, Aparicio, Pina, Valdés, & Arroyo, 2013), communities (Anacker & Harrison, 2012), or lineages radiated in particular hotspots (Valente et al., 2010). These approaches have permitted analysis of the effect of environmental factors in promoting differentiation and diversity and thus the building up of such hotspots (see Anacker, Whittall, Goldberg, & Harrison, 2011 for the California hotspot).

The Mediterranean region is one of the largest biodiversity hotspots in the world (Myers, Mittermeier, Mittermeier, da Fonseca, & Kent, 2000), further composed by a number of subhotspots. One of them lies in the Betic‐Rifean area (Médail & Quézel, 1997) and harbors a range of altitudes, lithologies, and climatic conditions that mostly reproduces those present in the whole Mediterranean Basin (Molina‐Venegas et al., 2013). In addition to many endemic plants locally evolved, it also harbors many refuged taxa that avoided extinction during drier and colder periods in the Miocene and the Pleistocene, respectively (Médail & Diadema, 2009; Rodríguez‐Sánchez & Arroyo, 2011). Within this area, Sierra Nevada is the core of the Betic‐Rifean hotspot and shows one of the highest floristic diversities in the Iberian Peninsula and around the Mediterranean Basin (Médail & Diadema, 2009; Molina‐Venegas et al., 2013). Very recently, its woody flora has been subjected to an evolutionary account of biodiversity using barcoding techniques and the construction of megaphylogenies, and it has been possible to determine the effects of elevation and substrate mosaicism on the phylogenetic structure of its species assemblages (Molina‐Venegas, Aparicio, Lavergne, & Arroyo, 2015; Simón‐Porcar et al., 2018). Whereas these studies have allowed detecting the prevailing evolutionary patterns, further insight requires scaling down to particular lineages diversified in the region, and even to species and population level of taxa subjected to systematic discussion, to disentangle how diversity in this hotspot is created and maintained, and the drivers involved. In this regard, unraveling the relationships between ecological, functional traits and genetic diversity of narrow endemic plants provide opportunities for understanding how evolutionary processes operate over these local spatial scales (Arafeh et al., 2002; Simón‐Porcar et al., 2018).

Large reviews of genetic data have provided valuable insights into the patterns of genetic variation and their correlates, showing their dependence on many factors related to geographic range, effective population size, life form, mating and breeding system, environmental changes and biogeographical events, demographic processes and history of populations, polyploidization, hybridization, and natural selection (Ellegren & Galtier, 2016). Nevertheless, it is also necessary to underline the existence of a strong phylogenetic signal in the levels of genetic diversity between related species due to the conservativeness of functional traits in evolution, particularly in pollen and seed dispersal mechanisms (Duminil et al., 2007). Overall, it could be expected that these factors act synergistically in biodiversity hotspots as drivers of genetic diversity and diversification.

The genus *Helianthemun* (Cistaceae) is a monophyletic lineage with about 136 species and subspecies characterized by a complex taxonomy and remarkable life history and functional trait diversity (Agulló, Pérez‐Bañón, Crespo, & Juan, 2015; Aragón & Escudero, 2008; Herrera, 1992; López‐González, 1993; Rodríguez‐Pérez, 2005). Originated in the Miocene, the genus diversified during the Pliocene and Pleistocene giving rise to three large radiating lineages, one diversified across the Saharo‐Arabian and Irano‐Turanian regions (sect. *Eriocarpum*), the other two diversified around the Mediterranean Basin and the Eurosiberian mountains (sections *Helianthemum* and *Pseudocistus*, respectively; Aparicio et al., 2017; Martín‐Hernanz et al., unpublished). Within these lineages, it is remarkable that currently most species have restricted ranges or are endemic to very small regions (cf., López‐González, 1993; Proctor & Heywood, 1968), whereas only some species have large geographic distribution areas (e.g., *H. apenninum*,* H. cinereum*,* H. kahiricum*,* H. ledifolium*,* H. lippii*,* H. nummularium*,* H. oelandicum*,* H. salicifolium,* or *H. stipulatum*).

This study focuses on four taxa of *Helianthemum* from Sierra Nevada (southern Spain) that are two pairs of relatives with disparate distribution area (two local endemics vs. two widespread) and soil preferences (two dolomite specialists vs. two soil generalist), which moreover represent two different lineages (subgenera) within *Helianthemum* (Aparicio et al., 2017). The aim of this study was to unravel the effect of environmental, reproductive, and phylogenetic factors on the patterns of genetic diversity and differentiation in our precise case study. Specifically, for each taxa, we have gathered data about soil characteristics, community composition, and floral traits (as subrogates of the breeding system) to assess the effect of these factors on their genetic diversity and spatial genetic distribution of nuclear DNA microsatellite variation. Beyond deriving possible implications for the implementation of conservation action plans for the two stenochorous taxa in the Sierra Nevada National Park involved in this study, the assessment of the microevolutionary forces that drive the species divergence and differentiation in *Helianthemum* in a context of recent radiation can further shed light on why many species in this genus are prone to endemism.

## 2 MATERIALS AND METHODS

### 2.1 Study area

Sierra Nevada is located at the core of the Betic‐Rifean region in SE Spain (37.07°N, 3.18°W) occupying an area of ca. 2,100 km^2^, the altitude ranging from 250 m a.s.l. to the highest peak in the Iberian Peninsula, the Mulhacén, at 3,482 m a.s.l. The climate is Mediterranean, characterized by cold winters and hot summers with a marked summer drought (July to August). The annual average temperature decreases in altitude from 12 to 16°C below 1,500 m to 0°C above 3,000 m a.s.l., and the annual average precipitation is about 600 mm, ranging from less than 250 mm in the lowest parts of the mountain range to more than 700 mm in summit areas. The number of vascular‐plant taxa recorded in Sierra Nevada is 2,353 (Lorite, 2016), about 12% restricted to the Betic mountains (Blanca et al., 2002) and c. 80 local endemics (Lorite, Navarro, & Valle, 2007). This mountain range is currently considered one of the most important biodiversity hotspots in the Mediterranean region (Médail & Diadema, 2009) benefitting from several legal protections such as Biosphere Reserve MAB Committee UNESCO, Special Protection Area, and Site of Community Importance (Natura 2000 network) and National Park.

### 2.2 Study taxa and sampling

**Figure 1:**
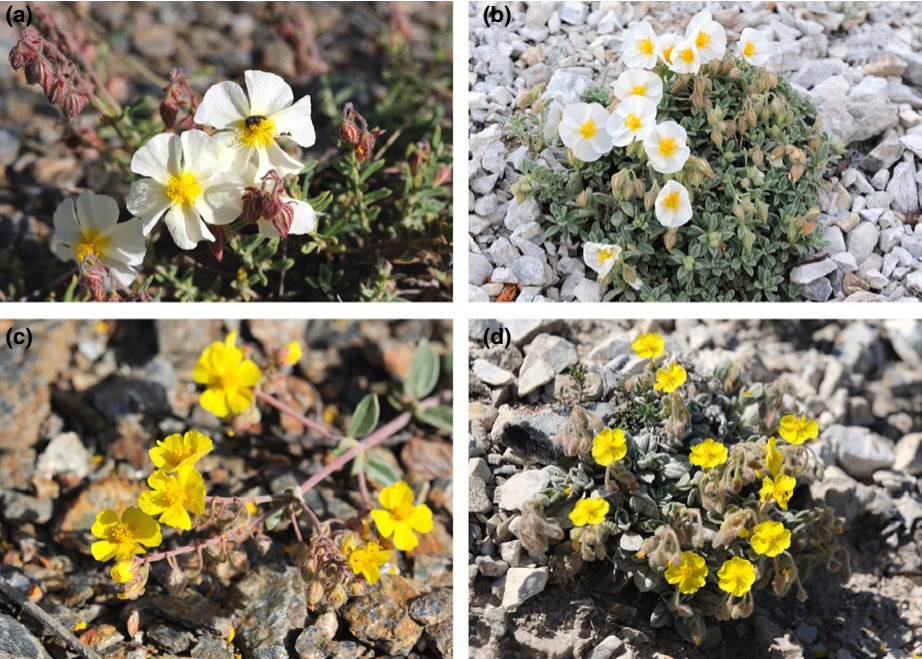
Studied taxa: (a) *Helianthemum apenninum* (L.) Miller subsp. *apenninum*; (b) *H. apenninum* subsp. *estevei* (Peinado & Mart. Parras) G. López; (c) *H. cinereum* subsp. *rotundifolium* (Dunal) Greuter; (d) *H. pannosum* Boiss. Nomenclature follows López‐González (1993)

The four study taxa are chamaephytic shrubs belonging to the genus *Helianthemum* (Cistaceae; Figure 1): *H. pannosum* and *H. cinereum* subsp. *rotundifolium* (sect. *Pseudocistus*, subg. *Plectolobum*) and *H. apenninum* subsp. *estevei* and *H. apenninum* subsp. *apenninum* (sect. *Helianthemum*, subg. *Helianthemum*; nomenclature follows López‐González, 1993). *Helianthemum apenninum* subsp. *estevei* and *H. pannosum* are stenochorous endemic taxa (ETs) restricted to dolomitic soils in Sierra Nevada, whereas *Helianthemum apenninum* subsp. *apenninum* and *H. cinereum* subsp*. rotundifolium* (hereafter referred as *H. cinereum*) are mostly sympatric taxa (WTs) distributed in C and W of the Mediterranean region with preference for limestone outcrops (Table 1). Nevertheless, it is necessary to stress that these WTs can never be found cohabiting with the former congeneric ETs. Full or partial self‐incompatibility has been reported in the few perennial species of *Helianthemum* whose breeding system have been studied (Tébar, Gil, & Llorens, 1997; Rodríguez‐Pérez, 2005; Aragón & Escudero, 2008; Agulló et al., 2015; but see Alonso et al., 2013 who considered *H. cinereum* to be self‐compatible) and, although the pollination biology of most species remains unknown, flowers are visited by generalist insects, mostly bees and beetles (Proctor, 1956; Rodríguez‐Pérez, 2005; Agulló et al., 2015; personal observations).

**Table 1:** Values (mean ± *SE*) of genetic diversity parameters computed through microsatellite loci Characterization of the four studied taxa of *Helianthemum*

Taxa	Subgenus	Distribution range	Soil preference	*N*	*Nloci*	*Na*	*Ne*	*He*	*Fis*
*H. apenninum* subsp. *apenninum*	*Helianthemum*	W Europe	Limestone	42	7	4.43 (±0.84)	1.76 (±0.27)	0.345 (±0.103)	0.458 (±0.146)
*H. apenninum* subsp. *estevei*	*Helianthemum*	Sierra Nevada	Dolomite	36	7	4.57 (±0.97)	2.23 (±0.38)	0.470 (±0.099)	0.396 (±0.162)
Two‐sample nonparametric studentized permutation test results						*p *=* *0.750	***p *** **=** *** *** **0.016**	***p *** **=** *** *** **0.016**	*p *=* *0.156
*H. cinereum* subsp. *rotundifolium*	*Plectolobum*	CW Mediterranean	Limestone	34	11	5.27 (±0.91)	2.80 (±0.70)	0.492 (±0.079)	0.103 (±0.157)
*H. pannosum*	*Plectolobum*	Sierra Nevada	Dolomite	28	8	5.25 (±1.07)	3.17 (±0.67)	0.593 (±0.084)	0.140 (±0.122)
Two‐sample nonparametric studentized permutation test results						*p *=* *0.266	*p *=* *0.656	*p *=* *0.547	*p *=* *0.930

Note

Sample size (*N*), number of polymorphic loci (*Nloci*), number of alleles (*Na*), effective number of alleles (*Ne*), unbiased expected heterozygosity (*He*), and inbreeding coefficient (*Fis*). Differences for genetic diversity parameters between widespread and endemic taxa within each subgenus assessed through two‐sample nonparametric studentized permutation tests. Significant values highlighted in bold.

**Figure 2:**
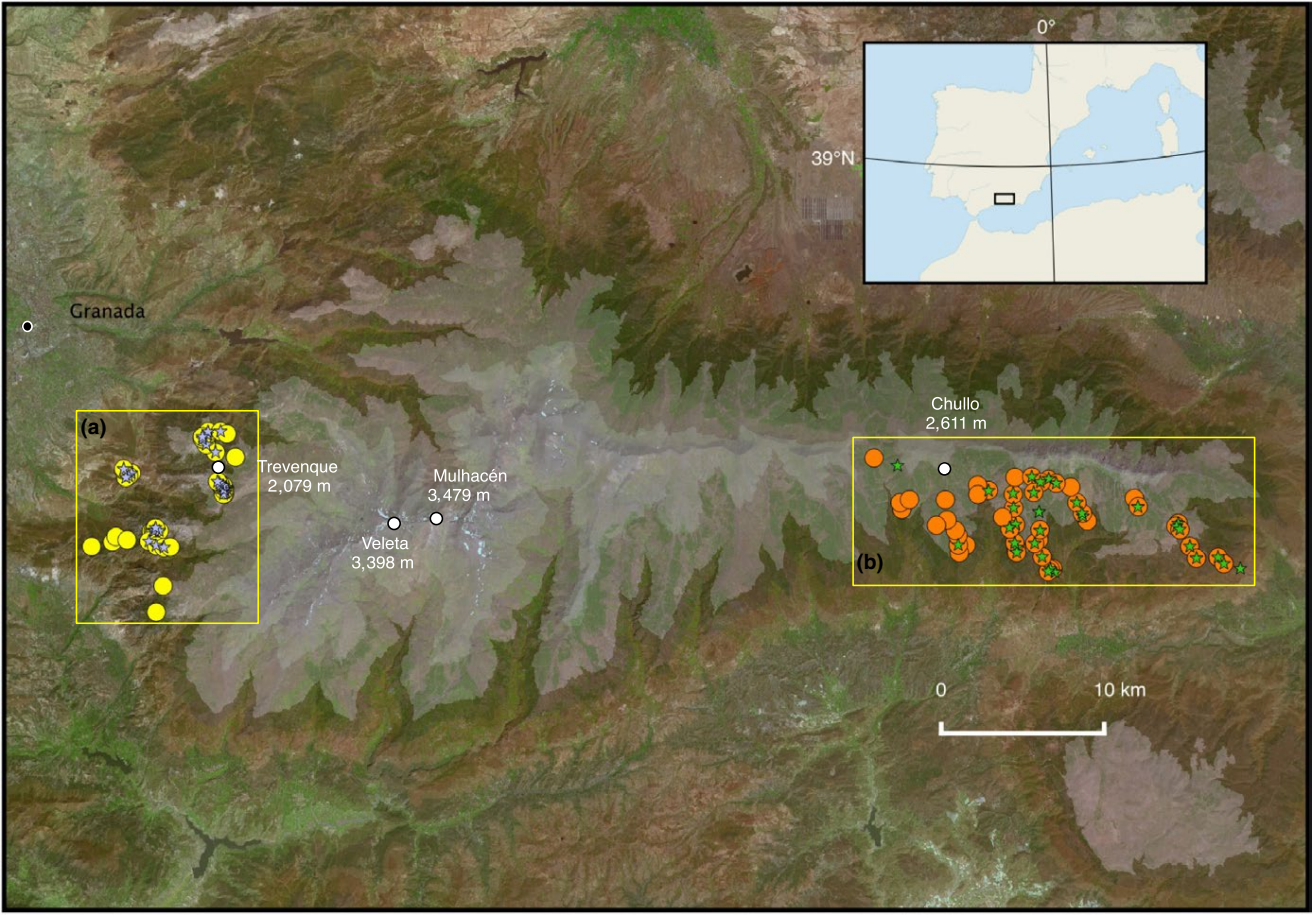
Study area and sample sites in Sierra Nevada National Park, southern Spain. (a), massive dolomite outcrop showing sample points of *Helianthemum pannosum* (blue stars) and *H. apenninum* subsp*. estevei* (yellow circles). (b), limestone (plus mica‐schist) outcrop showing sample points of *H. cinereum* subsp*. rotundifolium* (green stars) and *H. apenninum* subsp. *apenninum* (orange circles). *Helianthemum pannosum* and *H. apenninum* subsp*. estevei* are restricted to dolomite outcrops in Sierra Nevada, whereas *H. cinereum* subsp*. rotundifolium* and *H. apenninum* subsp. *apenninum* are more or less widely distributed from the Iberian Peninsula to Greece and the Maghreb

In Sierra Nevada, we selected two areas differing in soil type (Figure 2): (a) a massive dolomite outcrop about 132 km^2^; and (b) a limestone (plus mica‐schist) outcrop about 197 km^2^, where the ETs *Helianthemum apenninum* subsp. *estevei* and *H. pannosum* and the WTs *H. apenninum* subsp. *apenninum* and *H. cinereum* can be found more or less widely distributed, respectively, the interindividual distance of individual conspecific plants ranging from a few centimeters to a few kilometers. In this context, we followed a Trapper sampling scheme (Schwartz & McKelvey, 2009) because samples (i.e., individual plants) were randomly drawn across the sampling areas and explicitly discarded a population‐level approach because such approach could result in the overestimation of the number of genetic groups when analyzing the spatial genetic structure of individual plants (Schwartz & McKelvey, 2009). Here, we sampled a total of 140 individual plants between 1,260 and 2,230 m in altitude in early June 2014 (Table 1), and to avoid biases in the measures of genetic variation and structure we sampled all the four taxa in a similar area (i.e., 132 and 197 km^2^) and interindividual distance (cf. Cole, 2003). Whenever possible, depending on orographic circumstances, we aimed to keep the mean interindividual distance of the sampled conspecific plants ca. 5 km: 3.64 in *H. pannosum* (ranging 0.07–8.06), 5.95 in *H. cinereum* (ranging 0.08–17.34), 4.54 in *H. apenninum* subsp. *estevei* (ranging 0.05–11.79), and 6.25 in *H. apenninum* subsp. *apenninum* (ranging 0.22–22.39). Sampling included fresh leaves immediately dried in silica gel for further DNA extraction, plus 68 flowers and floral buds from 59 individual plants (Table 3) fixed in 70% ethanol then kept at 4°C until dissection for floral traits quantification. To cope with an eventual variation in reproductive outputs throughout the season (e.g., Yorke et al., 2011), all the flowers were collected the same day coinciding with the beginning of blooming period (Blanca et al., 2002; López‐González, 1993). We also collected the most recently opened flower and the next‐to‐open floral bud of the central inflorescences of each individual sample plant.

### 2.3 DNA isolation and microsatellite genotyping

We ground 30 mg of dehydrated leaf tissue with a Retsch MM200 shaker mill and isolated total genomic DNA from each specimen with the Invisorb Spin Plant HTS 96 Kit (Invitek, Berlin, Germany) according to the manufacturer's protocol. We checked for transferability to the other three studied taxa a set of 12 microsatellites primer pairs originally developed for *H. cinereum* subsp. *rotundifolium* (Molecular Ecology Resources Primer Development Consortium et al. 2013). We carried out PCR amplification reactions in 5× reaction buffer (Bioline, London, UK) containing dNTPs and MgCl_2_, in a final volume of 10 μl with c. 30 ng of template DNA, 1 U of My*Taq* Red polymerase (Bioline, London, UK), 0.5% of BSA, 0.2 μM of the reverse and the M13 universal primers (this latter labeled with FAM, NED, VIC, or PET to the 5′‐end), and 0.07 μM of the modified forward primer with the M13 primer sequence attached at its 5′‐end. We carried out polymerase chain reactions on a MyCycler^™^ Thermal Cycler (Bio‐Rad, Hercules, CA) following two PCR profiles published elsewhere (for details in cycle conditions see Molecular Ecology Resources Primer Development Consortium et al. 2013). Amplified products were analyzed on an ABI 3730 DNA Analyzer (PE Applied Biosystems, Foster City, CA, USA) at “Unidad de Genómica” (Universidad Complutense, Madrid, Spain). Microsatellite scoring was automatically done with the software GeneMapper v.3.7 (PE Applied Biosystems) and manually corrected when necessary.

### 2.4 Soil and environmental sampling

We also collected 125 soil samples at 10–20 cm depth close to each of the 140 sampled plants (except for plants occurring less than 5 m apart). These soil samples were left to dry for 24 hr at room temperature and then passed through a 2‐mm sieve before determining the following physicochemical parameters: texture (percentage of sand, silt and clay), pH (at 25°C 1:5), electrical conductivity (μS/cm, at 25°C 1:5), percentage of carbonates and organic matter, content of macronutrients (mg/kg of N‐Kjeldahl and P‐Olsen), micronutrients (mg/kg of Cu, Fe, Mn, Zn, and Bo), and assimilable cations (meq/100 g of Ca, Mg, K, and Na). Soil analyses were performed by “Laboratorio Agrama SL” (Seville, Spain).

We also recorded in the field the community neighborhood and composition of each individual sampled plant by recording (a) the number of conspecifics in a 5‐m radius buffer, (b) the distance to the nearest conspecific plant, and (c) the percentage of shrub coverage also in a 5‐m radius buffer following a visual scale with four categories: 1 = 1%–25%, 2 = 26%–50%, 3 = 51%–75%, and 4 = 76%–100%.

### 2.5 Floral traits

For each taxon, we averaged the number of stamens and ovules per ovary after dissecting one floral bud from 5 to 21 individual plants under a dissecting microscope totaling 59 flower buds (Table 3). For each sample, we also estimated the number of pollen grains per anther by squashing one anther randomly selected on a slide containing Isoton II (Beckman Coulter, Fullerton, CA) and counting the number of pollen grains contained in aliquots of 500 μl using a particle counter (Coulter Multisizer 3, Beckman Coulter). Pollen production per flower was estimated as the number of pollen grains per anther (mean value of three aliquots) multiplied by the number of anthers in the flower; then, we computed the pollen‐to‐ovule (P/O) ratios accordingly (Cruden, 1977). We also dissected 1–3 recently‐opened flowers from 7 to 10 individual plants per taxon to measure: (a) length of petals, (b) height of the tallest anther, (c) height of the ovary, and (d) height of the stigma; then, we computed the stigma‐anther separation (i.e., herkogamy) by subtracting the height of the tallest anther from the height of the stigma (ovary size plus style length). Stigma‐anther separation has positive values when stamens do not reach the stigma height (i.e., approach herkogamy) and negative values when the stigma is below the anthers (i.e., reverse herkogamy).

### 2.6 Data analysis

#### 2.6.1 Genetic diversity and inference of historical events on genetic structure

We estimated the following standard parameters of genetic diversity: observed number of alleles (*Na*), effective number of alleles (*Ne*), unbiased Nei's gene diversity (*He*), and inbreeding coefficient (*Fis*) with GenAlEx 6.5 (Peakall & Smouse, 2012). We averaged individual loci values to obtain values for each taxa. Departure from Hardy–Weinberg equilibrium for each locus in each taxa was assessed through chi‐squared tests with GenAlEx 6.5. Statistical differences in genetic diversity parameters between widespread taxa (WTs) and their endemic counterparts (ETs) were assessed through two‐sample nonparametric studentized permutation tests (10,000 permutations) for paired data with the package *nparcomp v.2.6* (Konietschke, Placzek, Schaarschmidt, & Hothorn, 2015) in R 3.3.3 (R Developmental Core Team, 2017).

To assess the number of independent genetic and evolutionary entities, we inferred the genetic structure by two methodologically contrasting approaches: the Bayesian clustering procedure of Structure (Hubisz, Falush, Stephens, & Pritchard, 2009; Pritchard, Stephens, & Donnelly, 2000) and the discriminant analysis of principal components (DAPC: Jombart, Devillard, & Balloux, 2010). Both methods allow to identify the optimal number of clusters (*K*) in the data set and to assign simultaneously the sampled individuals to each of the inferred clusters. Nonetheless, each of these methods has limitations that can affect the validity of their results. The Bayesian clustering methods infer subtle population structure by minimizing linkage disequilibrium and departures from Hardy–Weinberg equilibrium within each inferred cluster (Pritchard et al., 2000), which are often difficult to verify and can restrict their applicability. In fact, the assumption of panmixia does not necessarily hold in these taxa, which can exhibit a mixed‐mating system in natural populations (see Discussion). Alternatively, Principal Components Analysis (PCA)‐based methods construct low‐dimensional projections of the data that maximally retain the variance–covariance structure among the sample genotypes. However, while these low‐dimensional projections allow for straightforward visualization of the underlying population structure, it is not always straightforward to derive and interpret estimates for global ancestry of sample individuals from their projection coordinates (Novembre & Stephens, 2008). We restricted these analyses to the set of five microsatellite loci common to all four taxa (see Results) in order to avoid potential effects of the nonrandom distribution of missing data. Since the main aim of our study is to detect possible admixture between individuals of the four studied taxa, the number of potential *K* clusters assessed ranged from 1 (assuming a single panmictic entity) to 4 (assuming every taxon formed its own cluster). The Bayesian clustering was conducted in Structure 2.3.4 applying a series of five independent runs for each value of *K*. All runs consisted of 10^5^ iterations of burn‐in period plus a final run length of 10^6^ iterations, and convergence of Markov chains was assessed after the analyses. We assessed the optimal number of clusters with the procedure by Evanno, Regnaut, and Goudet (2005) and summarized the results of independent replicate runs and visualized them with the help of the online software Pophelper (Francis, 2017). The DAPC analysis was conducted with the R package *adegenet v.2.0.1* (Jombart, 2008). The optimal number of genetic clusters (*K*) was estimated with the function *find.cluster* and identified as the one with the lowest BIC value.

We used the program Bottleneck 1.2.02 (Cornuet & Luikart, 1996) to detect recent demographical changes in the effective population size of the study taxa. We employed Wilcoxon's tests to detect heterozygosity excess in comparison with simulated values under mutation‐drift equilibrium with two mutational models of microsatellite variation: the infinite allele model (IAM) and the two‐phase mutational model (TPM), the latter allowing for 10% of single‐step changes (Cristescu, Sherwin, Handasyde, Cahill, & Cooper, 2010). The variation rate was set to 12, as recommended by Piry, Luikart, and Cornuet (1999) for microsatellite markers.

#### 2.6.2 Environmental data

We computed pairwise genetic correlation coefficients for individuals of the same taxa and plotted average values against geographic distance (represented by eight distance classes: 0–1, 1–2, 2–4, 4–6, 6–8, 8–10, 10–15, 15–20 km). We assessed significance of the averaged correlation coefficients through the construction of 95% confidence intervals by randomly permuting individual location 1,000 times and constructed the correlograms with GenAlEx 6.5.

To assess significant relationships among environmental variables and genetic diversity for each taxa, we constructed a genetic distance (among individuals) matrix with GenAlEx 6.5 that was subsequently subjected to multiple regression on distance matrices (MRM) analyses against the following environmental matrices: (a) a “neighborhood–composition distance” computed as the pairwise Euclidean distance from the three measures (number of conspecifics, distance to the nearest neighbor and shrub cover) of neighborhood composition recorded in the field, (b) a “soil distance”, computed by applying a Principal Components Analysis to the standardized soil parameters and then retaining the first two principal components to calculate the pairwise Euclidean distance, and (c) a “geographic distance” computed as the pairwise Euclidean distance among each sampled individuals. All environmental matrices were computed with the function *dist* and the MRM analyses with the package *ecodist* (Goslee & Urban, 2007) in R.

#### 2.6.3 Floral traits

We assessed differences among taxa in the petal length, stigma‐anther separation, and the P/O ratio through general linear mixed models (GLMMs) with the package *lme4* (Bates, Maechler, Bolker, & Walker, 2015) in R. We set individuals as random factor to account for measures accomplished on different flowers of the same individual, log‐transformed P/O ratio before model adjustment, and validated models by checking the absence of significant patterns in the residuals of the fitted models. We accomplished significant differences among pairs of taxa through sequential Bonferroni correction.

## 3 RESULTS

### 3.1 Genetic diversity and inferred historical events

Genetic diversity values for the individual microsatellite loci amplified in the four *Helianthemum* taxa of this study are in Supporting Information Table S1. The 12 microsatellite loci originally developed for *H*. *cinereum* subsp. *rotundifolium* were partially transferable to the other three taxa with 11, 7, 7, and 8 loci yielding polymorphic amplified products in *Helianthemum cinereum* subsp. *rotundifolium*,* H*. *apenninum* subsp. *apenninum, H. apenninum* subsp. *estevei,* and *H. pannosum,* respectively (Supporting Information Table S1).

Genetic diversity measures (number of alleles, effective number of alleles, and the expected heterozygosity) were higher in the ETs (*H. apenninum* subsp. *estevei* and *H. pannosum*) than in the WTs (*H. apenninum* subsp. *apenninum* and *H. cinereum*, respectively), even significantly for some measures (Table 1). Inbreeding coefficients (*Fis*) were markedly different between subgenera with higher values in both taxa of subg. *Helianthemum* than in those belonging to subg. *Plectolobum*.

**Figure 3:**
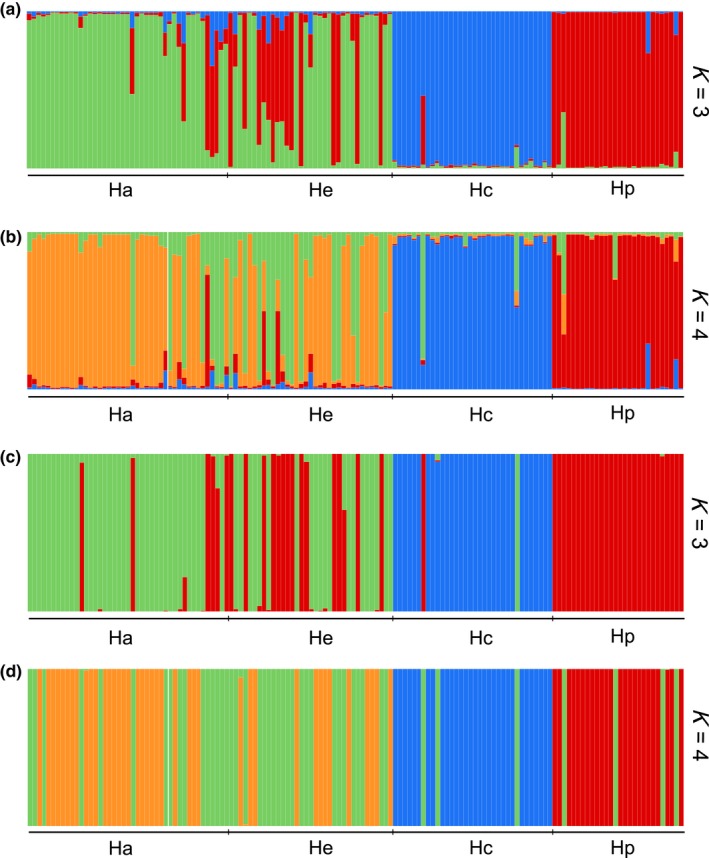
Genetic structure in the four taxa of *Helianthemum* studied: *H. apenninum* subsp. *apenninum* (Ha), *H. apenninum* subsp*. estevei* (He), *H. cinereum* subsp*. rotundifolium* (Hc), and *H. pannosum* (Hp) (44, 38, 34, and 28 individuals, respectively) as resolved by Bayesian clustering inferred by Structure at *K* = 3 (a) and *K* = 4 (b) and discriminant analysis of principal components (DAPC) at *K* = 3 (c) and *K* = 4 (d) of microsatellite markers, the two most likely number of clusters. Clusters inferred at each *K* are indicated by different colors. Each individual plant is represented by a vertical bar, colored proportionally according to the cluster assignment

The genetic structure inferred by Structure pointed to *K* = 3 as the most likely number of genetic groups followed by *K* = 4 (Figure 3), while the DAPC recovered *K* = 4 as the optimum number of clusters follow by *K* = 3. The genetic distinctiveness of *H. pannosum* and *H. cinereum* is apparent in both methods since nearly all individuals belonging to the two species of subgenus *Plectolobum* were differentiated into two dissimilar clusters associated with previously assumed species and irrespective of the number of *K*s and the analysis applied (Figure 3). However, the Bayesian analysis with the highest ∆*K* values for *K* = 3 considered the individuals from the two subspecies of *H. apenninum* as a single cluster. Results of the DAPC with the optimum number of clusters (*K* = 4) were not confirmed using the Bayesian Structure analysis because the cluster of *H. apenninum* was split into two. These two new clusters were not well defined because they had individuals from both subspecies indicating a labile genetic structure between them which can be explained by a very recent divergence or by the persistence of current gene flow. However, both subspecies can be considered as two different entities since the proportion of the individuals assigned to these two clusters were considerably asymmetric between both subspecies (*H. apenninum* subsp. *apenninum*: 27 individuals of the first cluster vs. 16 individuals of the second one; *H. apenninum* subsp. *estevei*: 14 individuals of the first cluster vs. 22 of the second one; Figure 3), and further, the two subspecies display clear ecological and morphological differentiation (López‐González, 1992, 1993; see Discussion).

The program Bottleneck detected significant heterozygosity excess in *H. pannosum* under the IAM model of microsatellite evolution (*p *=* *0.014), indicative of a recent population decline. We also detected a significant heterozygosity deficiency in *H. apenninum* subsp. *apenninum* under both the IAM and TPM models (*p *=* *0.039 and *p *=* *0.012, respectively) indicative of a recent population expansion. Either heterozygosity excess or deficiency was not detected in the other two taxa.

### 3.2 Environmental data

**Figure 4:**
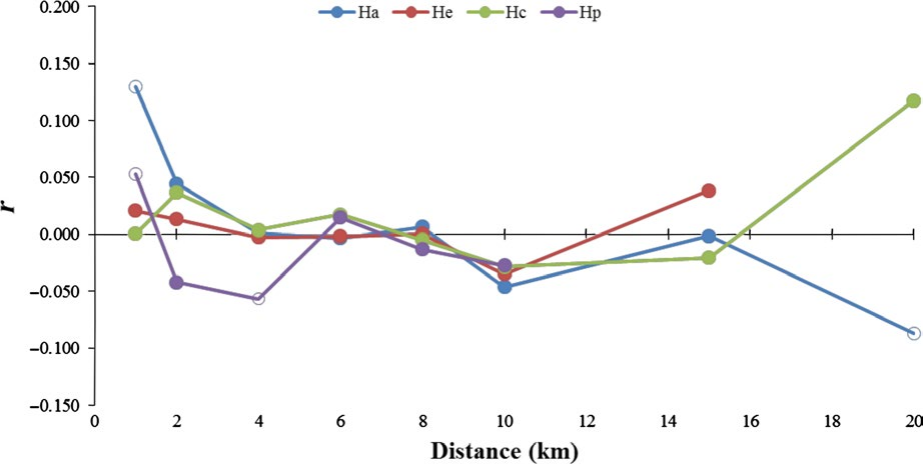
Spatial correlograms for the four taxa of *Helianthemum* studied: *H. apenninum* subsp. *apenninum* (Ha), *H. apenninum* subsp*. estevei* (He), *H. cinereum* subsp*. rotundifolium* (Hc), and *H. pannosum* (Hp). Empty symbols are correlation coefficients (*r*) significantly (*p *<* *0.05) different from the null hypothesis of random spatial structure based on permutations tests

Spatial correlograms showed different results among taxa (Figure 4). *Helianthemum apenninum* subsp*. apenninum* showed a significant and positive autocorrelation in the pairwise genetic coefficient at the first distance class (0–1 km), followed by a decline with significant but negative spatial autocorrelation coefficients above 20 km. *Helianthemum pannosum* showed a significant and positive autocorrelation in the pairwise genetic coefficient at the first distance class (0–1 km) and at 2–4 km. No significant spatial signal was detected in any distance class for the other two taxa. Results of multiple regressions on distance matrices are shown in Table 2. Genetic distance showed marginally significant correlation with soil parameters (all soil variables standardized) in *H. apenninum* subsp. *estevei* (*p*‐value = 0.054). The neighborhood–composition distance matrix and the geographic distance matrix were uncorrelated with genetic distance in all cases.

**Table 2:** Results of multiple regression matrices (MRM) analyses of the genetic distance matrices in the four studied taxa of *Helianthemum* against the soil, neighborhood–composition, and the geographic distance matrices

Taxa	*F*	*p*‐value
*H. apenninum* subsp. *apenninum*		
Neighborhood	2.58E‐03	0.771
Soil	−1.05E‐01	0.465
Geographic distance	1.50E‐02	0.733
*H. apenninum* subsp. *estevei*		
Neighborhood	−8.35E‐03	0.279
**Soil**	**2.68E‐01**	**0.054**
Geographic distance	3.26E‐05	0.508
*H. cinereum*subsp. *rotundifolium*		
Neighborhood	1.48E‐03	0.215
Soil	−2.29E‐01	0.293
Geographic distance	1.27E‐04	0.293
*H. pannosum*		
Neighborhood	−1.08E‐02	0.371
Soil	1.98E‐01	0.229
Geographic distance	7.57E‐05	0.290

Note

Marginally significant effects are highlighted in boldface (0.06 < *p*‐value < 0.05).

### 3.3 Floral traits

**Figure 5:**
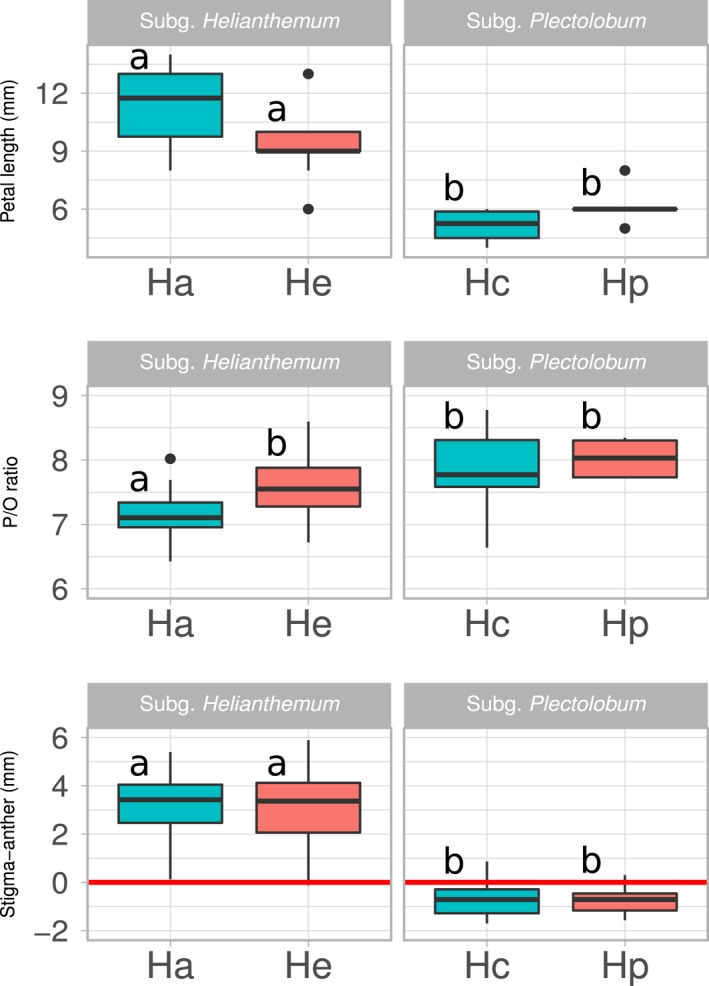
Boxplots for (a) petal length, (b) pollen‐to‐ovule (P/O) ratio, and (c) stigma‐anther separation (herkogamy) for the four taxa of *Helianthemum* studied: *H. apenninum* subsp. *apenninum* (Ha), *H. apenninum* subsp*. estevei* (He), *H. cinereum* subsp*. rotundifolium* (Hc), and *H. pannosum* (Hp). The boxplot displays the smallest and largest values as well as the first quartile, the median, and the third quartile. Outliers are indicated with dots. Different letters indicate statistical significant differences among taxa after a sequential Bonferroni correction on regression coefficients from GLMM models

The two taxa of subg. *Helianthemum* (*H. apenninum* subsp. *apenninum* and *H. apenninum* subsp. *estevei*) had on average flowers with petals about 10 mm in length, 20 ovules per ovary, high pollen production per flower, low pollen‐to‐ovule ratio, and approach herkogamy, whereas those of subg. *Plectolobum* (*H. cinereum* and *H. pannosum*) had almost constantly six ovules per ovary, smaller petals of about 5–6 mm, lower pollen production, higher pollen‐to‐ovule ratio, and reverse herkogamy (Table 3). Significant differences were found between petal length of the two subgenera and P/O ratio of *H. apenninum* subsp. *apenninum* and the other three taxa (Figure 5). However, the ETs showed lower average values in the number of stamens and ovules and higher average values of P/O ratio (albeit no statistically significant) than their corresponding WTs relatives (Table 3, Figure 5).

**Table 3:** Mean values (± *SE*) of petal length, number of stamens, number of ovules, number of pollen grains per flower, pollen‐to‐ovule ratio (P/O), tallest stamen height, stigma height (ovary size plus style length), and stigma‐anther separation measured for the study taxa of *Helianthemum*:* H. apenninum*subsp. *apenninum* (Ha), *H. apenninum*subsp*. estevei* (He), *H. cinereum*subsp*. rotundifolium* (Hc), and *H. pannosum* (Hp)

Taxa	Petal length (mm)	Number of individuals	Num. of stamens	Num. of ovules	Pollen production per flower	P/O ratio	Tallest anther height (mm)	Stigma height (mm)	Stigma‐anther separation (mm)
Ha	11.46 ± 3.31 (12)	21	66.4 ± 1.9 (21)	23.4 ± 1.3 (21)	28,755 ± 2,314 (21)	1,277 ± 130 (21)	7.59 ± 2.19 (12)	10.64 ± 3.07 (12)	3.05 ± 0.45 (12)
He	9.33 ± 2.33 (16)	12	60.9 ± 3.1 (12)	19.7 ± 2.2 (12)	38,220 ± 4,270 (12)	2,264 ± 326 (12)	6.16 ± 1.54 (16)	9.41 ± 2.35 (16)	3.05 ± 0.43 (16)
Hc	5.11 ± 1.11 (21)	21	58.2 ± 2.2 (21)	6.1 ± 0.3 (21)	16,760 ± 1,636 (21)	2,840 ± 379 (21)	4.31 ± 0.94 (21)	3.60 ± 0.79 (21)	−1.40 ± 0.14 (21)
Hp	6.33 ± 2.00 (10)	10	31.3 ± 4.6 (5)	5.5 ± 0.2 (5)	17,569 ± 7,209 (5)	2,957 ± 1,322 (5)	5.04 ± 1.59 (10)	4.27 ± 1.35 (10)	−0.77 ± 0.18 (10)

Note

Sample size (number of flowers) in brackets.

## 4 DISCUSSION

Our integrated study of a set of factors that can be either directly or indirectly predictors for genetic diversity (distribution range, phylogenetic relationships, habitat type, and floral traits) of two pairs of narrow/widespread congeners of *Helianthemum* showed somewhat unexpected results. In particular, (a) the narrow endemic study taxa (ETs) did not show lower values of genetic diversity but even higher, as well as higher average values of pollen production per flower and pollen‐to‐ovule ratio than their widespread relatives (WTs), and (b) the two taxa of subg. *Helianthemum*, with larger corollas, positive stigma‐anther separation (i.e., approach herkogamy) and higher pollen production than the two taxa of subg. *Plectolobum*, displayed lower genetic diversity and higher values of inbreeding. These results disclose that genetic diversity may indeed be affected by a large number of factors, some of them acting in opposite directions, and that patterns of genetic diversity cannot be readily predicted from simple proxies (Arafeh et al., 2002). On the other hand, the fact that we have found that mating system rather than geographic distribution is driving the differences in genetic variability between the two pairs of relatives in this study and the existence of a (albeit marginally) significant effect of the substrate profile in the genetic variability of the dolomite specialist *H. apenninum* subsp. *estevei* may be further indicating the important role of these factors on the diversification and species differentiation in *Helianthemum*, also showing that proneness to endemism in the genus is associated with ecological specialization, given the apparent distinct distribution and habitat types of many endemics (López‐González, 1992). Altogether, from a conservation biology perspective our results underline that management and conservation action plans relying only on simple proxies for genetic diversity such as rarity, geographic range, successful sexual reproduction, or outbreeding may be masking the actual genetic patterns.

### 4.1 Genetic diversity and historical events

In this study, we have found that genetic diversity of the ETs (*H. pannosum* and *H. apenninum* subsp. *estevei*) was at least equal or higher than in the WTs (*H. cinereum* and *H. apenninum* subsp. *apenninum*; Table 1). It is usually assumed that rare species tend to have low levels of genetic variability due to the small population size (e.g., Arafeh et al., 2002; Edwards, Lindsay, Bailey, & Lance, 2014; Turchetto et al., 2016). However, small population size coupled with substantial genetic diversity is not unusual in plants and has been reported for several rare species (Barrett & Kohn, 1991; Ellstrand & Elam, 1993; Lutz, Schneller, & Holderegger, 2000). Therefore, range extension on its own is not a reliable predictor of genetic diversity (Premoli, Souto, Allnutt, & Newton, 2001) and we must invoke alternative explanations to explain the unexpected high levels of genetic diversity found in the ETs.

Historical changes in population size and long‐term isolation have often been proposed as possible explanations for such a lack of correlation between population size and genetic diversity (e.g., Landergott, Holderegger, Kozlowski, & Schneller, 2001). For example, the high genetic diversity found in small populations of some alpine species (also in the study area of Sierra Nevada) such as *Gentiana alpina*,* Kernera saxatilis,* and *Papaver alpinum* has been linked to shifts in their distribution ranges and vicariance processes occurred during the Pleistocene (Kropf, Comes, & Kadereit, 2006). In our case, the high genetic diversity found in *H. pannosum*, which seems to have suffered a recent population decline according to the significant heterozygosity excess that we have detected (see above), can be the outcome of the successful retention of genetic variation in current populations after the expansion–contraction cycles occurred during the Pleistocene climatic changes and historical population bottlenecks. Contrarily, the lower genetic diversity of *H. apenninum* subsp. *apenninum* compared with its narrow endemic relative *H. apenninum* subsp. *estevei* can be consequence of a recent expansion favored by the postglacial conditions as it has been described in other widespread and probably young species of *Helianthemum* such as *H. nummularium* and *H. oelandicum* (Soubani, 2010; Soubani, Hedrén, & Widén, 2015; Volkova, Schanzer, Soubani, Meschersky, & Widén, 2016).

Alternatively, the high levels of genetic diversity that we have found in the ETs could be due to the existence of gene flow between the related taxa (Smith & Voung‐Pham, 1996) and to recent diversification or phylogenetic constraints. The genetic distinctiveness of *H. pannosum* and *H. cinereum* detected by Structure and DAPC (see Figure 3) led us to discard contemporary hybridization events between the two species of subg. *Plectolobum*. However, the fact that individuals from the two subspecies of *H. apenninum* were considered as a single cluster by Structure or integrated into two not well‐defined clusters by DAPC could indicate a recent divergence with persistence of gene flow between both subspecies. This is expected because the diversification of subg. *Helianthemum* did not likely occur until the Pleistocene (Aparicio et al., 2017) and the reproductive barriers between their members may be still labile, particularly at infraspecific levels (Nieto‐Feliner, 2014). Although the taxonomic splitting of species subjected to systematic discussion such as, for example, *H. apenninun*,* H. nummularium*,* H. marifolium*,* H. cinereum,* or *H. oelandicum* into species and subspecies has been widely criticized because diagnostic characters are not stable but correlated with ecological conditions in most of the cases (Soubani et al., 2015; Volkova et al., 2016; Widén, 2015, 2018); indeed, this seems not to be the case of *H. apenninum* subsp. *apenninum* and *H. apenninum* subsp. *estevei*, two taxonomic entities that beyond clear ecological differentiation show disparate morphological features (*H. apenninum* subsp. *estevei* is a shiny woody plant covered with long silky hairs on the surface of sepals and leaves, whereas *H. apenninum* subsp. *apenninum* is a greenish plant lacking any kind of long silky hairs (López‐González, 1992, 1993).

Other narrow endemic taxa in the genus *Helianthemum* are also characterized by high genetic diversity as exemplified by *H. gonzalezferreri*,* H. juliae,* and *H. inaguae* in the Canary Islands, which despite their very limited distribution range and population size, are all characterized by high genetic variation at the species level (González‐Pérez, Batista, & Sosa, 2013; González‐Pérez, Polifrone, Marrero‐Gómez, Bañares, & Sosa, 2015; Santana‐López, 2015). All these species have been considered relicts of ancient larger populations and wider distribution, but further studies specifically addressing these issues are required.

### 4.2 Genetic diversity and environmental data

*Helianthemum apenninum* subsp*. apenninum* and *H. pannosum* showed a significant and positive spatial autocorrelation in the first distance class (0–1 km; Figure 4), an isolation‐by‐distance pattern usually interpreted to be consequence of limited dispersal, in agreement with the prevalent gravity dispersal mechanism reported for some species of *Helianthemum* (González‐Pérez et al., 2013; Tébar et al., 1997). However, the absence of the isolation‐by‐distance pattern in *H*. *apenninum* subsp. *estevei* (Table 2; Figure 4) and the (marginally) significant effect of the substrate profile in the genetic variability are suggestive of the presence of geographic or ecological barriers to gene flow imposed by the high lithological specificity and the patchy distribution of the dolomitic outcrops. Moreover, this result can indicate the existence of active mechanisms of adaptation to the stressful conditions since dolomitic soils drain much more efficiently promoting strong xericity (Mota et al., 2008; Salmerón‐Sánchez et al., 2014). Despite the usual view regarding endemic taxa to be local specialists with reduced genetic variation (e.g., Thompson, 2005), numerous studies have shown that adaptation to stressing soil conditions can promote diversification within lineages eventually leading to increased levels of diversity at different spatial scales (Molina‐Venegas et al., 2015; Rajakaruna et al., 2003), which is congruent with the higher levels of genetic diversity retrieved for the dolomite specialist *H. apenninum* subsp. *estevei*.

The prevalence of endemic species is outstanding in some Mediterranean taxonomic groups (Thompson, 2005), as is also noticeable in *Helianthemum*. This relative abundance of endemic taxa in a particular lineage can be consequence of adaptation to different soil conditions, as discussed, coupled with the lack of mechanisms for long‐distance dispersal. Indeed, it is worth mentioning that many species of *Helianthemum* are soil specialists thriving exclusively on specific substrates such as gypsum (e.g., *H. squamatum*,* H. alypoides*,* H. marifolium* subsp. *conquense*), sandy‐soils (e.g., *H. guerrae*,* H. marminorense*), saline‐soils (*H. polygonoides*), or dolomite (e.g., *H. marifolium* subsp. *frigidulum*,* H. pannosum*,* H. apenninum* subsp. *estevei, H. viscidulum*,* H. neopiliferum*; López‐González, 1992; Sánchez‐Gómez, Carrión, & Carrión‐Vilches, 2001). This tendency suggests that the ability to adapt to special substrates is a driver for speciation in *Helianthemum*, albeit soil‐stress tolerance could simply be a more general adaptation to arid environments (Salmerón‐Sánchez et al., 2014). Furthermore, it has been suggested the effect of heterogeneous soil conditions promoting selective pressure on life history traits (i.e., variation in flowering strategies and variation in indumentum) in some other *Helianthemum* species (Soubani, 2010). However, specific and more detailed studies based on genetic mapping and field experiments should be applied to test whether the effect of the substrate profile in the genetic variability in *H. apenninum* subsp. *estevei* is the result of (a) spatial structuring due to the constraints imposed by soil conditions; or (b) the existence of local adaptation (Savolainen, Lascouz, & Merilä, 2013).

### 4.3 Genetic diversity and floral traits

Perianth size, stigma‐anther separation, P/O ratio, or life form are biological traits usually considered to be indicative of mating and breeding systems (Barrett, 2003; Cruden, 1977), also associated with range size (Lavergne, Thompson, Garnier, & Debussche, 2004). For instance, narrowly distributed species may exhibit reproductive traits prone to reduce outcrossing compared to their widespread relatives (e.g., fewer and smaller flowers, less stigma‐anther separation, and lower pollen‐ovule ratios) as a strategy for persistence after local adaptation (Lavergne et al., 2004). Contrary to expectations, we have found in this study that *H. pannosum* and *H. apenninum* subsp. *estevei* (i.e., the ETs) had higher pollen production per flower and higher P/O ratios than their widespread relatives (*H. cinereum* and *H. apenninum* subsp. *apenninum*). This result is likely showing that variation in floral traits is common in *Helianthemum*, even within populations (Aragón & Escudero, 2008; Martín‐Hernanz et al., unpublished), and the prevalence of mixed‐mating systems in these species (Aragón & Escudero, 2008; Rodríguez‐Pérez, 2005). Moreover, since *H. pannosum* and *H. apenninum* subsp. *estevei* also displayed a higher number of alleles (*Na*), effective number of alleles (*Ne*), and unbiased expected heterozygosity (*He*), it seems that mixed‐mating systems are promoting survival and contributing to the maintenance, or even to the increase, of the genetic diversity of these two stenochorous taxa. This striking result has also been documented in the Cretan endemic *Cyclamen creticum*, whose genetic variability is not lower than in its widespread relative *C. repandum*, being the levels of genetic diversity more influenced by the mating system rather than by the amplitude of the geographic distribution (Affre & Thompson, 1997).

On the other hand, it is remarkable that the differences between the two lineages are stronger than between ETs and WTs. The two taxa of subg. *Helianthemum* have shown floral traits usually considered to characterize outcrossing species (i.e., large petals, high number of stamens, ovules and pollen production per flower, high approach herkogamy; Martínez‐Peralta, Molina‐Freaner, Golubov, Vázquez‐Lobo, & Mandujano, 2014), whereas those of subg. *Plectolobum* showed floral features characterizing selfers (i.e., small flower size, low pollen production, and reverse herkogamy; Martínez‐Peralta et al., 2014). Also, and contrary to expectations, we have detected lower P/O values, lower values of effective number of alleles (*Ne*), and higher inbreeding coefficient (*Fis*) in both taxa of subg. *Helianthemum* than in those of subg. *Plectolobum* showing that they are more prone to selfing (Proctor, 1956; Widén, 1986; Tébar et al., 1997; Rodríguez‐Pérez, 2005; Aragón & Escudero, 2008; Agulló et al., 2015; but see Alonso et al., 2013). Altogether, it seems clear that floral traits are more strongly determined by the phylogenetic relatedness than by local adaptive processes that run at a shorter time scale (Ornelas, Ordano, De‐Nova, Quintero, & Garland, 2007).

### 4.4 Conservation implications

We have found that in *Helianthemum*, a recently evolved lineage where many species are currently just differentiating (Aparicio et al., 2017), genetic diversity is actually being driven by many intrinsic and extrinsic factors that act synergistically in promoting diversification in Sierra Nevada. This finding stresses the essential role of robust and dated phylogenies embracing the species under study to interpret patterns and to develop action plans for locally evolved of refuged species in biodiversity hotspots. Nevertheless, the core question in conservation genetics about how does genetic variability compare among rare and their widespread related species still persists (Gitzendanner & Soltis, 2000; Turchetto et al., 2016).

The two strictly dolomitic endemics in Sierra Nevada here studied (*H. pannosum* and *H. apenninum* subsp. *estevei*) have been evaluated as Vulnerable by the Spanish Red List of Endangered Plants (Moreno, 2008) due to their restricted geographic distribution range, the low number of populations, and the impact by grazing animals, albeit the rarity of these plants is probably due to natural causes related to the ecological specificity and the scarcity and the natural fragmentation of their habitats (Blanca et al., 2002). In our study, we have found that facultative xenogamy and mechanisms of adaptation to the stressful conditions imposed by dolomite soils can stand for the persistence on the study taxa (see Table 1 and Table 3); however, if we take into account the global warming scenario these populations, at the verge of alpine vegetation, can be at risk of rapid extinction because the Mediterranean summits are even at higher risk compared to other European mountains (Parmesan, 2006; Pauli et al., 2012). Therefore, *H. pannosum* and *H. apenninum* subsp. *estevei* require ongoing monitoring of their conservation status and it would be advisable the development of appropriate conservation programs to integrate both in situ and ex situ conservation techniques (Volis & Blecher, 2010). One key ex situ strategy for biodiversity conservation is the implementation of germplasm collections which have to be designed aiming to capture as much as genetic diversity as possible, especially regarding the rarest alleles (Caujapé‐Castells & Pedrola‐Monfort, 2004). The kind of precise genetic information at spatial scale that we provide in this study could be easily included in the implementation of germplasm collection programs by the environmental managers to maximize their cost‐effectiveness and to ensure the conservation of endangered species.

## AUTHOR CONTRIBUTIONS

The idea and design of the study were developed by R.G.A., J.A., and A.A. Plant material was collected by S.M.S., R.G.A., J.L., J.A., and A.A. The analyses and interpretation of the data were carried out by S.M.H., S.M.S, and R.G.A. Finally, the manuscript was written and discussed between all authors but was led by S.M.H. and A.A.

## Data Availability

Microsatellite data can be found at Dryad Digital Repository: doi.org/10.5061/dryad.221v1f0/1
