# Supplementary material for: Genetic diversity and differentiation in narrow versus widespread taxa of Helianthemum (Cistaceae) in a hotspot: The role of geographic range, habitat, and reproductive traits
Source: Ecol Evol. 2019 Mar 5;9(6):3016–29. doi: 10.1002/ece3.4481 (PMC6434548; doi:10.1002/ece3.4481)
Supplement: Supplementary file 1 [file ECE3-9-3016-s001.docx]

Table S1. Genetic diversity values for the individual microsatellite loci amplified in the four *Helianthemum* taxa of study. Number of alleles (*Na*), effective number of alleles (*Ne*), unbiased expected heterozygosity (*He*) and inbreeding coefficient (*Fis*).

| Locus | *Na* | *Ne* | *He* | *Fis*^a^ |
| --- | --- | --- | --- | --- |
| *H. apenninum* subsp. *apenninum* |  |  |  |  |
| Helian_6 | 8 | 1.766 | 0.440 | 0.751^**^ |
| Helian_7 | 3 | 1.081 | 0.076 | -0.031^ns^ |
| Helian_8 | 6 | 2.840 | 0.657 | 0.374^**^ |
| Helian_16 | 5 | 2.404 | 0.596 | 0.452^**^ |
| Helian_17 | 5 | 2.087 | 0.528 | 0.671^**^ |
| Helian_20 | 2 | 1.025 | 0.024 | -0.012^ns^ |
| Helian_24 | 2 | 1.100 | 0.092 | 1.000^**^ |
| *H. apenninum* subsp. *estevei* |  |  |  |  |
| Helian_6 | 8 | 2.963 | 0.675 | 0.665^**^ |
| Helian_7 | 2 | 1.508 | 0.342 | -0.273^ns^ |
| Helian_8 | 5 | 2.618 | 0.631 | 0.326^**^ |
| Helian_16 | 6 | 3.879 | 0.765 | 0.524^**^ |
| Helian_17 | 7 | 2.228 | 0.565 | 0.546^**^ |
| Helian_20 | 2 | 1.029 | 0.029 | -0.014^ns^ |
| Helian_24 | 2 | 1.385 | 0.282 | 1.000^**^ |
| *H. cinereum* subsp. *rotundifolium* |  |  |  |  |
| Helian_2 | 5 | 3.150 | 0.694 | 0.646^**^ |
| Helian_3 | 4 | 1.244 | 0.199 | 0.227^**^ |
| Helian_4 | 13 | 9.242 | 0.907 | 0.613^**^ |
| Helian_6 | 6 | 3.939 | 0.758 | 0.188^ns^ |
| Helian_7 | 4 | 2.143 | 0.541 | -0.544^*^ |
| Helian_8 | 2 | 1.973 | 0.500 | -0.789^**^ |
| Helian_10 | 5 | 1.299 | 0.234 | 0.845^**^ |
| Helian_16 | 4 | 2.037 | 0.517 | -0.309^**^ |
| Helian_17 | 8 | 3.292 | 0.708 | -0.139^**^ |
| Helian_20 | 3 | 1.144 | 0.128 | -0.057^ns^ |
| Helian_24 | 4 | 1.285 | 0.225 | 0.453^**^ |
| *H. pannosum* |  |  |  |  |
| Helian_2 | 3 | 1.278 | 0.222 | 0.265^ns^ |
| Helian_4 | 10 | 7.113 | 0.880 | 0.612^**^ |
| Helian_6 | 9 | 4.226 | 0.778 | -0.067^*^ |
| Helian_7 | 3 | 2.142 | 0.543 | -0.474^**^ |
| Helian_10 | 7 | 3.453 | 0.725 | 0.099^**^ |
| Helian_16 | 4 | 3.213 | 0.703 | 0.535^**^ |
| Helian_17 | 4 | 2.608 | 0.628 | 0.039^ns^ |
| Helian_20 | 2 | 1.352 | 0.265 | 0.114^ns^ |

^a^ Deviance from Hardy-Weinberg equilibrium after chi-squared tests: ^ns^ = not significant, ^*^ *P* < 0.05, ^**^ P < 0.001.
